# Geologic Drivers of Late Ordovician Faunal Change in Laurentia: Investigating Links between Tectonics, Speciation, and Biotic Invasions

**DOI:** 10.1371/journal.pone.0068353

**Published:** 2013-07-15

**Authors:** David F. Wright, Alycia L. Stigall

**Affiliations:** 1 School of Earth Sciences, the Ohio State University, Columbus, Ohio, United States of America; 2 Department of Geological Sciences and OHIO Center for Ecology and Evolutionary Studies, Ohio University, Athens, Ohio, United States of America; Ludwig-Maximilians-Universität München, Germany

## Abstract

Geologic process, including tectonics and global climate change, profoundly impact the evolution of life because they have the propensity to facilitate episodes of biogeographic differentiation and influence patterns of speciation. We investigate causal links between a dramatic faunal turnover and two dominant geologic processes operating within Laurentia during the Late Ordovician: the Taconian Orogeny and GICE related global cooling. We utilize a novel approach for elucidating the relationship between biotic and geologic changes using a time-stratigraphic, species-level evolutionary framework for articulated brachiopods from North America. Phylogenetic biogeographic analyses indicate a fundamental shift in speciation mode—from a vicariance to dispersal dominated macroevolutionary regime—across the boundary between the Sandbian to Katian Stages. This boundary also corresponds to the onset of renewed intensification of tectonic activity and mountain building, the development of an upwelling zone that introduced cool, nutrient-rich waters into the epieric seas of eastern Laurentia, and the GICE isotopic excursion. The synchronicity of these dramatic geologic, oceanographic, and macroevolutionary changes supports the influence of geologic events on biological evolution. Together, the renewed tectonic activity and oceanographic changes facilitated fundamental changes in habitat structure in eastern North America that reduced opportunities for isolation and vicariance. They also facilitated regional biotic dispersal of taxa that led to the subsequent establishment of extrabasinal (=invasive) species and may have led to a suppression of speciation within Laurentian faunas. Phylogenetic biogeographic analysis further indicates that the Richmondian Invasion was a multidirectional regional invasion event that involved taxa immigrating into the Cincinnati region from basins located near the continental margins and within the continental interior.

## Introduction

Late Ordovician (~450 Ma) shallow marine deposits of Laurentia record a series of dramatic faunal and biogeographic shifts including a rapid change from predominantly warm to cool water adapted taxa during the middle Mohawkian (late Sandbian) and a major regional biotic immigration event into the Cincinnati Basin during the early Richmondian (Katian) [1,2]. These regional turnover events may have been driven by ongoing geologic processes such as the Taconian Orogeny or fluctuations in paleoceanographic conditions linked to global cooling; however, the precise relationship between evolutionary and geologic events has not been adequately investigated. In this analysis, phylogenetic biogeography, a rigorous, quantitative method, is employed within a species-level evolutionary framework to ascertain detailed patterns of biogeographic change and to illuminate the interaction between geologic processes and macroevolutionary patterns during the Late Ordovician in Laurentia. Specifically, we elucidate the relative impact of oceanographic vs. tectonic processes on biogeographic differentiation and assess their macroevolutionary impact.

### Synchronized Geologic and Biotic Change

**Figure 1:**
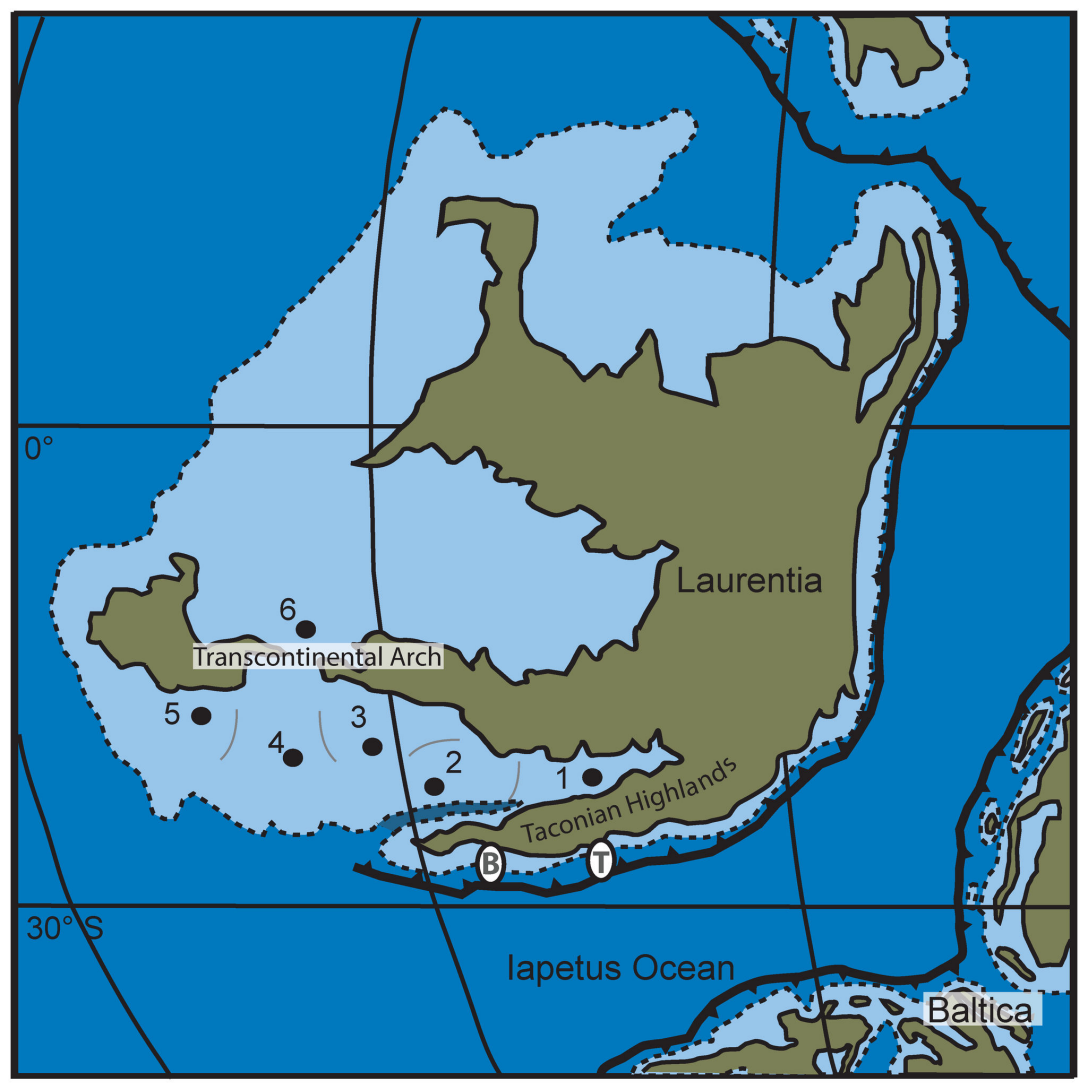
Late Ordovician paleogeography of Laurentia. Biogeographic areas analyzed are: (1), Anticosti Island (2);, Appalachian Basin (3);, Cincinnati Basin (4);, Central Basin (5);, Southern Midcontinent; and (6), Northern Midcontinent. Paleogeographic reconstructions (e.g. [45]) support the separation of these areas by physical barriers, such as structural platforms, trenches, and intracratonic arches, indicated with grey lines. Primary locus of tectonic activity during the Blountian (B) and Taconic (T) tectophases are indicated along the subduction zone.

**Figure 2:**
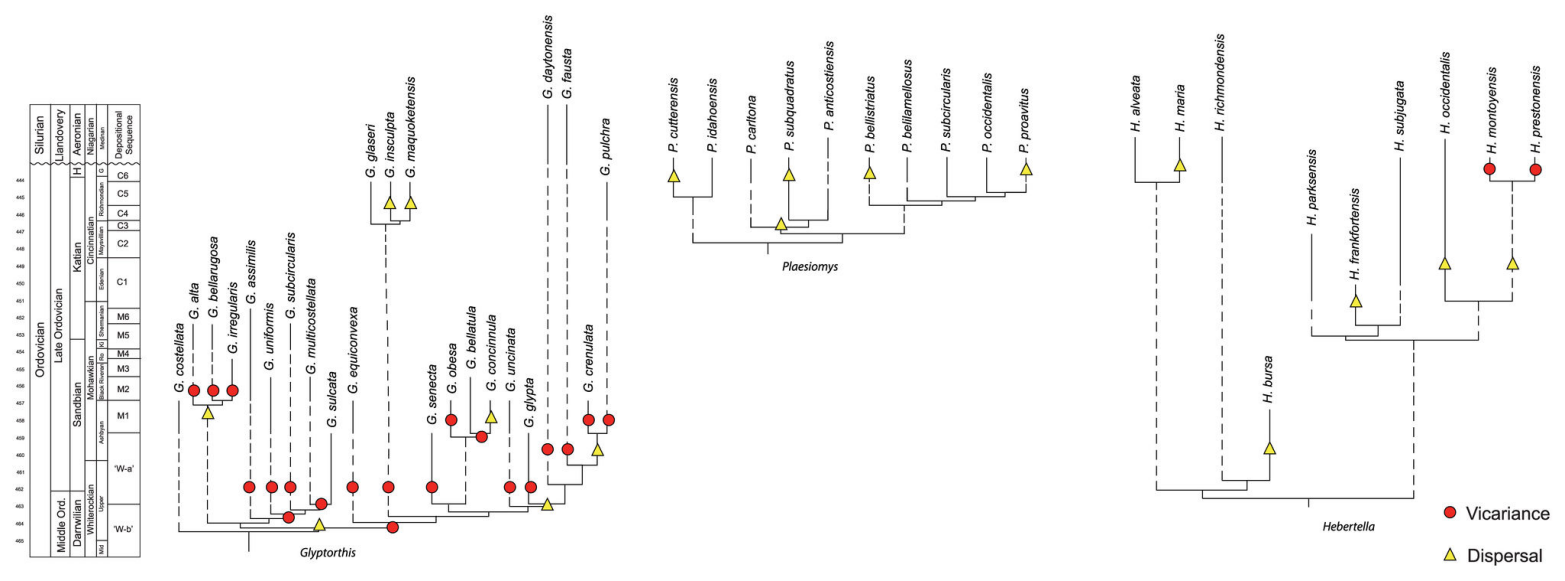
Stratocladograms. Species-level evolutionary and biogeographic patterns for *Glyptorthis* Foerste, 1914 [28], *Plaesiomys* Hall and Clarke, 1892 [29], and *Hebertella* Hall and Clarke 1892 [29] mapped onto a stratigraphic column at the level of depositional sequence. Ghost lineages reconstructed via equal phylogenetic split. Abbreviations: Ro, Rocklandian; Ki, Kirkfieldian; G, Gamachian; H, Hirnantian. Timescale modified from Holland & Patzkowsky [6].

During the Late Ordovician, the Taconian orogeny developed along the eastern margin of Laurentia as a result of diachronous collisions with an island arc and/or microplates [3–5] (Figure 1). The Taconian orogeny began with the Blountian tectophase along the extreme southeast margin of Laurentia during the Sandbian Stage [4,5]. During the Blountian tectophase, tropical carbonate deposition was widespread throughout Laurentia, and biogeographic and faunal divisions were relatively stable [5,6]. Following a brief quiescent interval, the locus of tectonic activity shifted northward to the New York promontory during the Taconic tectophase of the late Sandbian to Katian Stages [3,4]. The onset of the Taconic tectophase occurred during the middle Mohawkian (M5 sequence, late Sandbian, Figure 2) [4,5]. The associated crustal flexure, regional transgression, and siliciclastic influx coeval with an episode of global cooling introduced major lithologic and faunal changes throughout Laurentia.

Beginning in the middle Mohawkian (M5 sequence), carbonate deposits of eastern Laurentia reflect a heterozoan assemblage typified by cool water and/or non-photosynthetic organisms, whereas tropical-style carbonates became restricted to the continental interior and marginal basins [6,7]. This shift in carbonate deposition exhibits both regional and global geological signatures. Tectonic restructuring associated with the Taconic tectophase may have facilitated increased subsidence, thermohaline circulation, upwelling, and nutrient flux [5,8,9]. In addition, the mid-M5 sequence Guttenberg carbon isotope excursion (GICE) records a significant global cooling event corresponding to an episode of pre-Hirnantian Late Ordovician glaciation [10]. The synergistic combination of flexural downwarping and nutrient flux associated with the Taconic tectophase and coeval global cooling facilitated the development of an upwelling zone originating from the Sebree Trough which introduced cool, nutrient rich water into eastern Laurentia [5–7].

Also during the M5 sequence, faunas of the Appalachian Basin transitioned from predominantly warm to cool water adapted. The biotic overturn was accomplished primarily through immigration of extra-basinal taxa into a region following regional generic extinction [2]. For example, trilobites and some brachiopods interpreted as adapted to cooler water conditions participated in northward biogeographic expansion in associated with the spread of temperate carbonates [11,12].

The Richmondian Stage (late Katian [C4-C6 sequences], Figure 2) marked a return to tropical-style carbonate deposition across Laurentia which continued until the Hirnantian Stage. Whether the return to tropical conditions was due to a brief interval of global warming related to the Boda Event [13] or termination of upwelling due to basinal infilling [6] remains uncertain. Nonetheless, the C3/C4 sequence boundary corresponds to the return of tropical-style carbonate deposition in eastern Laurentia and marks the initiation of a large scale cross-faunal immigration event into the Cincinnati region known as the Richmondian Invasion [1,14,15].

The paleoequatorial region of the western United States and Canada has long been considered the biogeographic source of the Richmondian invaders [14,16]. However, this generalization has not previously been tested by detailed quantitative analysis and therefore requires further investigation because there were many possible source regions with different pathways of invasion available during the Late Ordovician (e.g., [17]). Invader taxa were geographically widespread throughout the Late Ordovician. Some invader genera simultaneously occupied multiple basins during the Richmondian Stage, such as Wyoming [18], Canada [19], and Tennessee [20]. The spatiotemporal pervasiveness of invasive genera limits the biogeographic precision of studies limited to genus-level patterns because a single invading species may belong to a genus present in multiple biogeographic areas. Consequently, a species-level phylogenetic framework, such as the one provided herein, is required to rigorously test competing hypotheses regarding the biogeographic sources of the Richmondian invaders and elucidate the geologic controls on Late Ordovician paleobiogeographic patterns.

## Materials and Methods

Phylogenetic biogeography is a powerful method for elucidating macroevolutionary and paleobiogeographic phenomena using the fossil record [21–24]. This analysis focuses on species of orthid brachiopods, which were among the most abundant organisms in Ordovician seas. Their benthic, sessile lifestyle makes them amenable to paleobiogeographic methods because inferred paleobiogeographic distributions of such species are likely to reflect actual distributions [25]. Species-level phylogenetic hypotheses [26,27] utilized include 53 species in three genera: *Glyptorthis* Foerste, 1914 [28]; *Plaesiomys* Hall and Clark, 1892 [29]; and *Hebertella* Hall and Clarke, 1892 [29]. These taxa were distributed among the tectonic basins of Laurentia during the late Middle to Late Ordovician (Figures 1-2). Two species, *Glyptorthis* *insculpta* (Hall, 1847) [30] and *Plaesiomys* *subquadratus* (Hall, 1847) [30], participated in the Richmondian Invasion. Each genus includes several species that are not incorporated within the phylogenetic framework due to inadequate preservation for character analysis (see discussion in [26,27]). *Hebertella* occurs in the Laurentian region of the British Isles [27]. *Plaesiomys* has been reported from Greenland [12]. *Glyptorthis* includes additional species in Gondwana and Baltica [26]. Each of these regions includes three or fewer species of the focal taxa. Regions with so few taxa are unable to be resolved within a phylogenetic biogeographic analysis [22]. Therefore, excluding these species from the data set does not alter the results of the analysis.

**Figure 3:**
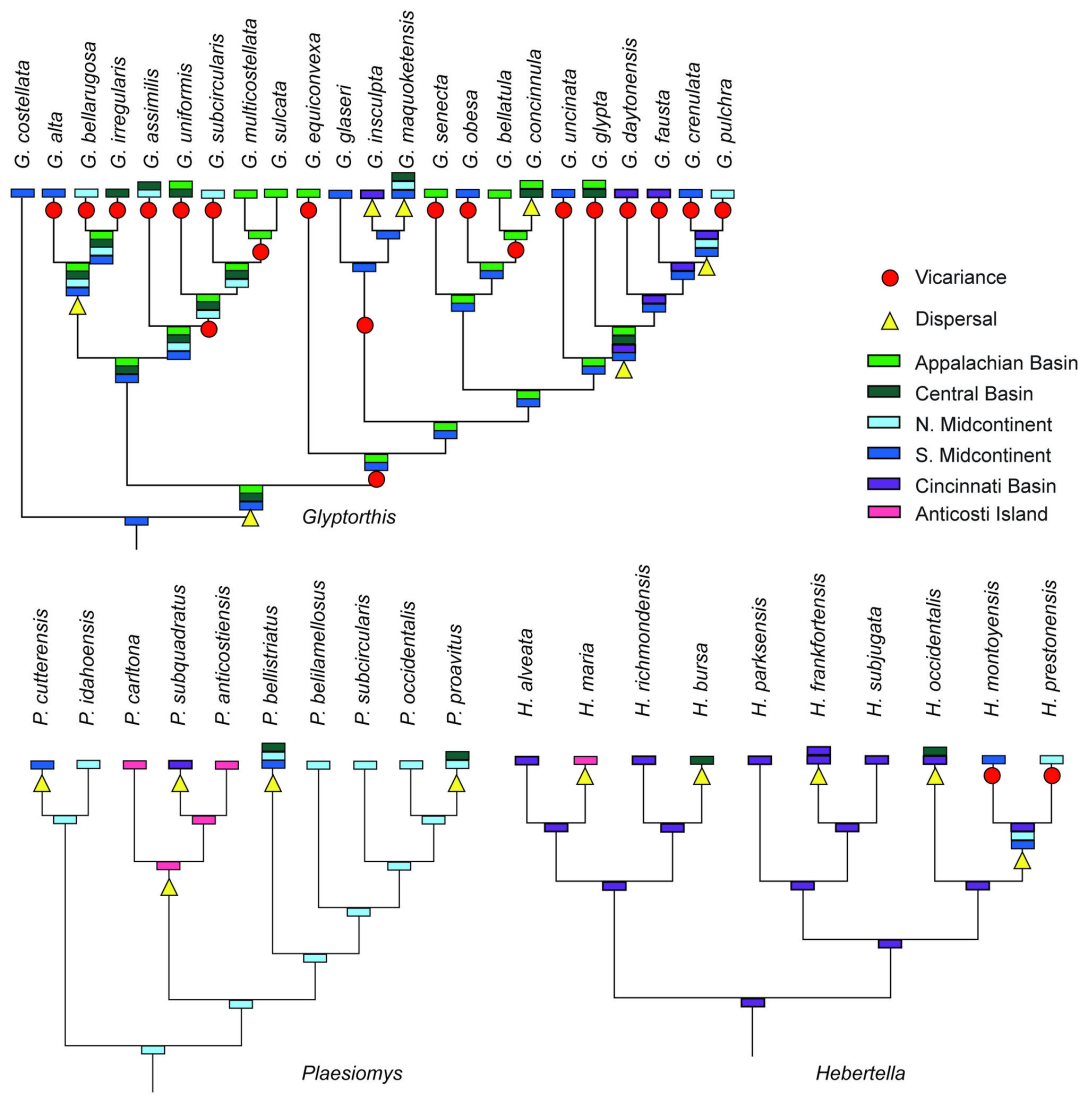
Taxon area cladograms. Cladograms depicting evolutionary and biogeographic relationships for species in *Glyptorthis* Foerste, 1914 [28], *Plaesiomys* Hall and Clarke, 1892 [29], and *Hebertella* Hall and Clarke 1892 [29]. Phylogenetic topology from Wright & Stigall [26,27]. Geographic distributions for terminal taxa were compiled from literature sources and museum collections. Only occurrence data for which the species could be visually verified (e.g., photo plate, museum specimen examined) were incorporated. Biogeographic states for ancestral nodes were optimized using Fitch Parsimony as described in Lieberman [22].

A Lieberman-modified Brooks Parsimony Analysis [LBPA] was employed as described in Wiley & Lieberman [31]. Species-level phylogenetic hypotheses were converted to taxon-area cladograms by placing the geographic distributions of each species at the tips of each cladogram and optimizing ancestral biogeographic areas onto the internal nodes using Fitch Parsimony (Figure 3). Shifts in reconstructed geographic distributions at cladogenetic events were examined to identify episodes of speciation by vicariance or dispersal. Episodes of biogeographic range contraction resulting in speciation events where a descendent species occupied a subset of the geographic range inhabited by its ancestor were identified as vicariance events; whereas speciation events associated with an episode of biogeographic range expansion in which the descendent species occupied geographic regions additional to those of its ancestor were identified as dispersal (Figure 3) [19,31].

**Figure 4:**
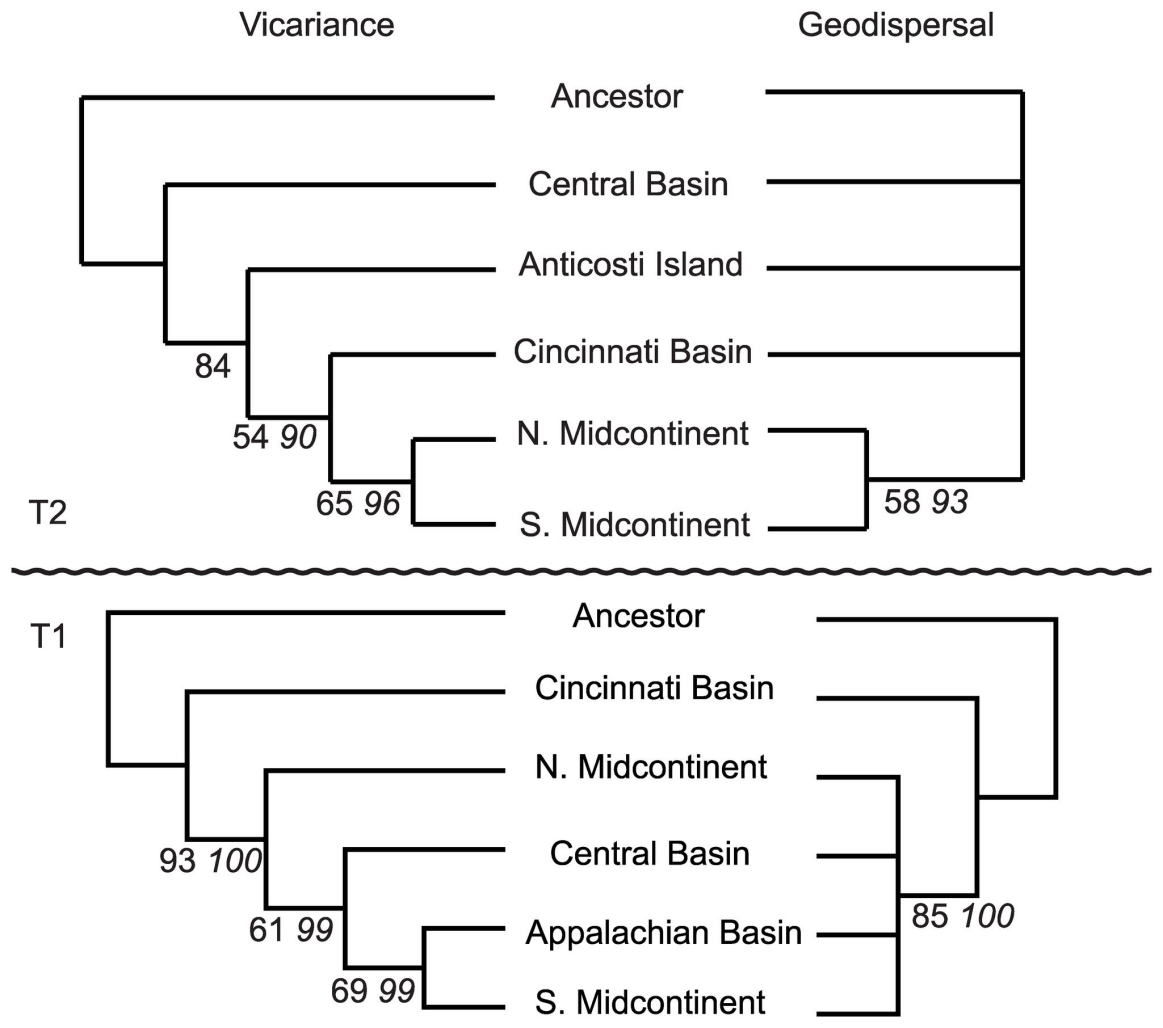
Vicariance and Geodispersal general area cladograms. The vicariance trees indicate the order that areas became separated from each other due to barrier (tectonic, eustatic, etc.) formation; whereas geodispersal trees indicate the relative order in which barriers were removed. Congruence between tree topologies indicates that geographic barriers rose and fell in the same relative order, suggesting the importance of cyclical geologic processes (e.g., oscillating sea levels). Conversely, incongruence indicates that singular events (e.g. tectonic pulses) influenced the evolution and biogeographic differentiation of the clades [22]. Numerical values indicate bootstrap (plain text) and jackknife (italicized) support for nodes. T1 analysis recovered a single most parsimonious vicariance tree (length of 87 steps, consistency index 0.736) and two most parsimonious geodispersal trees (64 steps, CI 0.797). T2 analysis recovered a single most parsimonious vicariance tree (49 steps, CI 0.816) and two most parsimonious geodispersal trees (59 steps, CI 0.531). The strict consensus topology is illustrated for geodispersal analyses.

Because different geologic processes operated before and after the M4/M5 sequence boundary, LBPA was employed in a time-stratigraphic framework to more accurately discern patterns of biogeographic differentiation. Stratigraphic dimension was incorporated based on equal phylogenetic split using first and last appearances of species at the level of depositional sequence. Cladogenetic events were separated into Time Slice 1 (T1), approximately corresponding to the Blountian tectophase (W-b through M4 sequences), and Time Slice 2 (T2), approximately corresponding to the Taconic tectophase (M5 though C6 sequences) (Figure S1). Additional subdivision of T2 into pre-Richmondian and post-Richmondian time slices resulted in too few data for analysis. As both sub-intervals are dominated by dispersal speciation, the results obtained from their combined analysis are consistent with results from analyzing them separately. If a lineage was extant during both time slices, the speciation event was coded as occurring during T1 to avoid paraphily in the data set. Consequently, all lineages coded for TS2 were monophyletic. A similar approach was taken by [32]. Vicariance and geodispersal matrices were coded for each time slice using the biogeographic data in the taxon-area cladograms (Figure S1 Table S1). General area cladograms were constructed from each matrix using parsimony analysis (Figure 4).

## Results and Discussion

### Macroevolutionary Impact of Biogeographic Change

Results of these analyses indicate fundamental differences in biogeographic and macroevolutionary patterns between the T1 and T2 intervals (Table 1). Analysis of speciation mode at cladogenetic events (Figure 3) identified vicariance as the dominant mode of speciation during the T1 interval, operating in 77% of the 26 discernible speciation events; whereas only 15% of 13 discernible speciation events during the T2 interval were attributable to vicariance (Figure 2). This pattern arises from differences in evolutionary differentiation of the genera analyzed through time (Figure 3). The taxon-area cladogram for species of *Glyptorthis* indicates that the genus originated in the Southern Midcontinent region and subsequently underwent a major dispersal event into the Appalachian and Central Basins (Figure 3). Accordingly, most of the ancestral lineages within *Glyptorthis* are reconstructed to occupy multiple tectonic basins (Figure 3). Because the early evolutionary history of the clade is characterized by geographically widespread species, most speciation events during T1 were accomplished via range contractions that led to speciation by vicariance. In contrast, ancestral species of *Plaesiomys* and *Hebertella* occurred predominantly in a single tectonic basin (the Northern Midcontinent and Cincinnati Basin, respectively) with frequent episodes of speciation via dispersal into neighboring tectonic basins during T2 (Figure 3). Documentation of this pattern in additional taxa would be desirable, but no additional species-level phylogenetic hypotheses exist for contemporaneous Laurentian endemics. The recovered pattern of high vicariance during T1 followed by high dispersal during the T2 is congruent with patterns documented by other researchers in genus-level biogeographic analyses among a diverse suite of Laurentian clades including corals, graptolites, gastropods, bivalves, monoplacophorans, ‘inarticulate’ brachiopods, and trilobites [2,33–36]. Although these patterns were documented at a higher taxonomic level, the consistent signal across clades and taxa supports the interpretation that our recovered pattern cannot be attributed to sampling bias alone.

**Table 1:** Comparison of speciation mode by Time Slice.

	**Vicariance Events**	**Dispersal Events**
**TS2**	2 (15%)	11 (85%)
**TS1**	20 (77%)	6 (23%)

The diametrically opposed patterns of speciation and biogeographic differentiation between T1 and T2 reflect different biogeographic controls operating before and after the M4/M5 sequence boundary. This discrepancy is clearly illustrated by the divergent topology of vicariance trees between times slices and the dissimilarity of the geodispersal and vicariance trees within each time slice (Figure 4). The T1 vicariance tree is well resolved and indicates a strong biogeographic relationship between the Appalachian Basin and the Southern Midcontinent region. The numerous T1 vicariance events produce a topology supported by strong tree statistics (Figure 4). Conversely, the strict consensus of the T1 geodispersal tree is unresolved, which reflects conflicting biogeographic patterns among the few speciation events occurring via dispersal in T1 (compare Figures 2, 3). The T2 vicariance tree is also well resolved and supported despite the limited number of vicariance events during the T2 interval, which indicates little conflict amongst recovered patterns. Notably, the geodispersal tree in T2 is even more unresolved than in T1 although speciation occurred predominantly by dispersal during this interval. The lack of resolution arises from conflicting data regarding dispersal pathways during T2 (compare Figures 2, 3). When the two most parsimonious geodispersal trees are examined individually, one is consistent with a close relationship between the Cincinnati Basin and the Midcontinent regions (supported by biogeographic affinities of *G* *. insculpta* ), whereas the other tree is consistent with a more recent exchange of taxa between Anticosti Island and the Midcontinent region (supported by biogeographic affinities of *P* *. subquadratus* ). These data indicate that both dispersal pathways were operational during the Richmondian Invasion.

The level of congruence between vicariance and geodispersal trees is indicative of the style of geologic processes impacting macroevolutionary patterns [22]. Congruent trees support the influence cyclical geologic processes, such as cyclical sea level or climatic changes, in which barriers arose (causing vicariance) and fell (facilitating dispersal) in the same relative order. Incongruous trees support the primacy of singular geologic forcers, such as tectonics or linear climate change [22]. In this case, the dissimilar vicariance and geodispersal tree topologies indicate that cyclical processes were less important than singular processes in facilitating Late Ordovician faunal differentiation.

#### Linking geologic and biotic processes

During T1, the Blountian tectophase introduced siliciclastics into eastern Laurentia yet did not inhibit the production of tropical carbonates [5,6]. The high rate of vicariant speciation during the T1 corroborates a study by Miller and Mao [36], which found an increased rate of diversification for late Whiterockian to early Mohawkian (Sandbian) genera across multiple clades located in regions closely associated with Blountian phase orogenesis. The species-level evolutionary and biogeographic patterns presented here, therefore, support Miller and Mao’s [36] hypothesis that the high rates of diversification may be due to increased levels of habitat partitioning associated with tectonic activity. Localized tectonic pulses of the Blountian tectophase provided fluxes of siliciclastic sediment to eastern Laurentia which would have fragmented previously large biogeographic ranges into disjunct areas with divergent environmental or ecological conditions thereby promoting speciation by vicariance.

Dispersal became the dominant mode of speciation during the Taconic tectophase of the Taconian Orogeny (T2). The combination of flexural downwarping of the craton and GICE related oceanographic changes facilitated the upwelling of cool, nutrient rich water into the shallow epieric seas of eastern Laurentia [4,5,9]. This induced regional extirpation [2] and effectively shut off the tectonic driver of vicariant speciation by eliminating the previously fragmented habit for warm-water adapted taxa. A similar decline in vicariant speciation occurred following the removal of geologic barriers during the Late Devonian [37,38].

The shift in speciation regime across the M4/M5 boundary is significant because high rates of vicariance are typically associated with high rates of speciation, whereas high rates of dispersal are associated with reduced speciation [39]. The negative correlation between dispersal and speciation rates results partly from increased numbers of invasive species. Intervals with frequent interbasinal biotic invasions develop reduced biodiversity due to suppressed speciation rates [39–41]. The introduction of ecologically broad invasive species produces a competitive regime in which incipient species tend to be eliminated rather than succeed; this propensity culminates in speciation gap during the post-invasion interval [39–41]. Although speciation rates are not quantitatively addressed here, a sharp decrease in generic diversification during the T2 was found by Miller and Mao ( [36]: p. 306, their Figure 2) and reduced speciation was reported following the Richmondian Invasion by Stigall [1]. Similar biogeographic patterns and associated diversity drops have been described in other taxa, such as Laurentian corals [34] and graptolites [35]. Further, Elias [42] noted that previously diverse coral faunas became impoverished during the Richmondian and many genera participating in the Richmondian Invasion were monospecific. The correlation between the development of a less speciose fauna and a shift to a dispersal dominated regime combined with no appreciable surge in extinction supports a decrease in speciation rates for Laurentian taxa during the T2 interval.

The main pulse of the Richmondian Invasion into the Cincinnati Basin occurred during the C5 sequence [6]. The return to tropical conditions facilitated an influx of low diversity, warm water fauna with a high propensity for dispersal. Data presented here indicate that the Richmondian Invasion represents a localized manifestation of the more generalized phenomenon of interbasinal invasion occurring throughout the T2 interval. The biogeographic origin of the invader species included in this analysis implicates both the Southern Midcontinent and Anticosti Island as source regions. Generic patterns from other taxa provide further support for multiple pathways of invasion. For example, rugose corals and bryozoans have closer biogeographic affinities to the Northern Midcontinent region [43,44].

## Conclusions

Phylogenetic biogeographic analysis of species-level patterns in Middle to Late Ordovician brachiopods indicates a shift in biogeographic patterns and macroevolutionary dynamics between the Blountian and Taconic tectophases of the Taconian orogeny (T1 and T2 intervals). These patterns were driven largely by geologic processes related to the Taconian Orogeny and global cooling at the boundary between the Sandbian and Katian stage boundary (M4/M5 sequence boundary). Prior to the M5 sequence, tectonic activity associated with the Blountian tectophase produced increased habitat fragmentation in tropical carbonate environments, thereby facilitating high levels of vicariant speciation. In contrast, the post-M5 sequence interval corresponds to a shift in paleoceanographic conditions related to the combined effect of structural deformation from the Taconic tectophase and GICE related global cooling, whereby cool, nutrient rich water from the Sebree Trough was introduced into shallow intracratonic seas. Dispersal became the dominant form of speciation and the increased number of invasive species may have led to increased faunal homogenization between basins. High rates of dispersal in the T2 interval and a return to tropical conditions within the Cincinnati Basin facilitated the Richmondian Invasion into the Cincinnati Basin. Species-level phylogenetic biogeographic patterns suggest both intracratonic seas and marginal basins supplied invasive species into the Cincinnati Basin.

## Supporting Information

Figure S1 Stratocladograms from Figure 2 with nodes and tips labeled to correspond with coding used in LBPA. The division between TS1 and TS2 is indicated by the horizontal line.
(TIF)

Table S1 Vicariance and geodispersal data matrices generated for Lieberman-modified Brooks Parsimony Analysis of Time-slice 1 and Time-slice 2. Numbered columns refer to nodes and tips on the stratocladograms (Figure S1). Coding as in Wiley & Lieberman [31].
(PDF)
