# Supplementary figures and images for: Geologic Drivers of Late Ordovician Faunal Change in Laurentia: Investigating Links between Tectonics, Speciation, and Biotic Invasions
Source: PLoS One. 2013 Jul 15;8(7):e68353. doi: 10.1371/journal.pone.0068353 (PMC3711913; doi:10.1371/journal.pone.0068353)

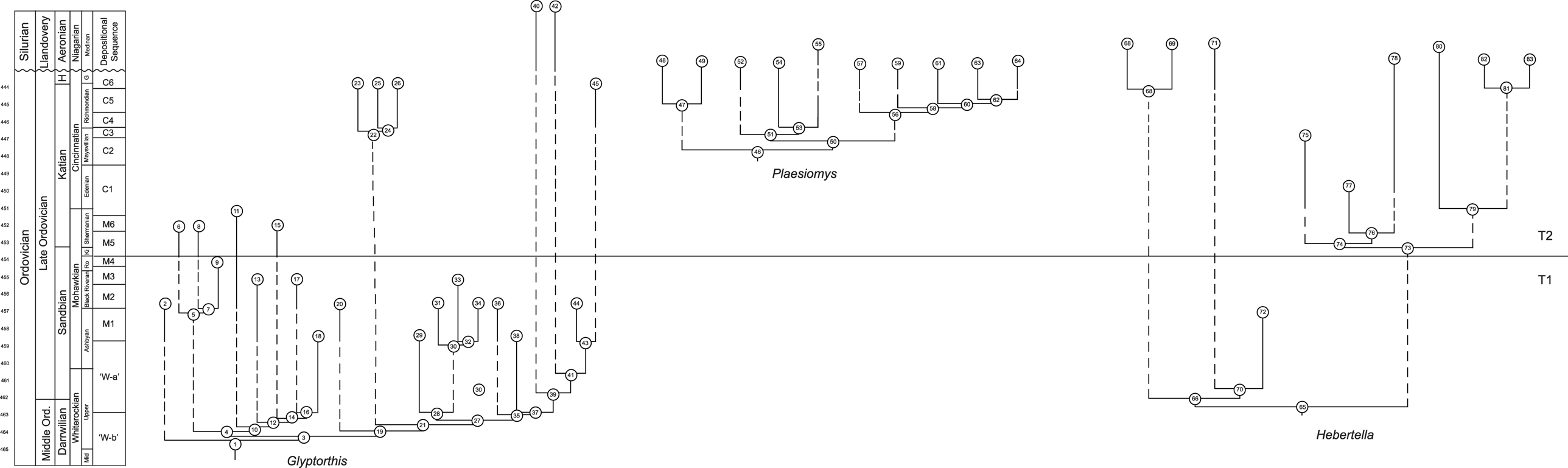

Supplement: Figure S1 — The division between TS1 and TS2 is indicated by the horizontal line. (TIF) [file pone.0068353.s001.tif]
