# Supplementary material for: Geologic Drivers of Late Ordovician Faunal Change in Laurentia: Investigating Links between Tectonics, Speciation, and Biotic Invasions
Source: PLoS One. 2013 Jul 15;8(7):e68353. doi: 10.1371/journal.pone.0068353 (PMC3711913; doi:10.1371/journal.pone.0068353)
Supplement: Table S1 — Numbered columns refer to nodes and tips on the stratocladograms (Figure S1). Coding as in Wiley & Lieberman [31]. (PDF) [file pone.0068353.s002.pdf]

Table S 1. Vicariance and geodispersal data matrices generated for LBPA of TS1 and TS2. Numbered columns refer to nodes and tips on the stratocladograms (Fig. S1). Coding as in [31].

TIME SLICE 1

Vicariance

	1	2	3	4	5	6	7	8	9	10	11	12	13	14	15	16	17	18	19	20	21	27	28	29	30	31	32	33	34	35	36	37	38	39	40	41	42	43	44	45	65	66	70	72
Ancestor	0	0	0	0	0	0	0	0	0	0	0	0	0	0	0	0	0	0	0	0	0	0	0	0	0	0	0	0	0	0	0	0	0	0	0	0	0	0	0	0	0	0	0	0
Appalachian Basin	0	0	1	1	1	1	1	1	1	1	1	2	2	1	1	2	1	1	2	2	1	1	1	2	1	1	2	1	1	1	1	1	2	1	0	0	0	0	0	0	0	0	0	0
Central Basin	0	0	1	1	1	1	1	1	2	1	2	2	2	1	1	1	0	0	1	0	0	0	0	0	0	0	0	0	1	0	0	1	2	1	0	0	0	0	0	0	0	0	0	1
Cincinnati Basin	0	0	0	0	0	0	0	0	0	0	0	0	0	0	0	0	0	0	0	0	0	0	0	0	0	0	0	0	0	0	0	1	1	2	2	1	2	1	1	1	1	1	1	1
N. Midcontinent	0	0	0	0	1	1	1	2	1	1	2	2	1	1	2	1	0	0	0	0	0	0	0	0	0	0	0	0	0	0	0	0	0	0	0	0	0	1	1	2	0	0	0	0
S. Midcontinent	1	1	1	1	1	2	1	1	1	1	1	1	0	0	0	0	0	0	2	1	1	1	1	1	1	2	1	0	0	1	2	1	1	2	1	1	1	1	2	1	0	0	0	0

Geodispersal

	1	2	3	4	5	6	7	8	9	10	11	12	13	14	15	16	17	18	19	20	21	27	28	29	30	31	32	33	34	35	36	37	38	39	40	41	42	43	44	45	65	66	70	72
Ancestor	0	0	0	0	0	0	0	0	0	0	0	0	0	0	0	0	0	0	0	0	0	0	0	0	0	0	0	0	0	0	0	0	0	0	0	0	0	0	0	0	0	0	0	0
Appalachian Basin	0	0	2	1	1	0	1	0	0	1	0	1	1	1	0	1	1	1	1	1	1	1	1	1	1	0	1	1	1	1	0	1	1	0	0	0	0	0	0	0	0	0	0	0
Central Basin	0	0	2	1	1	0	1	0	1	1	1	1	1	1	0	0	0	0	0	0	0	0	0	0	0	0	0	0	2	0	0	2	1	0	0	0	0	0	0	0	0	0	0	2
Cincinnati Basin	0	0	0	0	0	0	0	0	0	0	0	0	0	0	0	0	0	0	0	0	0	0	0	0	0	0	0	0	0	0	0	2	0	1	1	1	1	1	0	0	1	1	1	1
N. Midcontinent	0	0	0	0	2	0	1	1	0	2	1	1	0	1	1	0	0	0	0	0	0	0	0	0	0	0	0	0	0	0	0	0	0	0	0	0	0	2	0	1	0	0	0	0
S. Midcontinent	1	1	1	1	1	1	1	0	0	1	0	0	0	0	0	0	0	0	1	0	1	1	1	0	1	1	0	0	0	1	1	1	0	1	0	1	0	1	1	0	0	0	0	0

TIME SLICE 2

Vicariance

	22	23	24	25	26	46	47	48	49	50	51	52	53	54	55	56	57	58	59	60	61	62	63	64	67	68	69	73	74	75	76	77	78	79	80	81	82	83
Ancestor	0	0	0	0	0	0	0	0	0	0	0	0	0	0	0	0	0	0	0	0	0	0	0	0	0	0	0	0	0	0	0	0	0	0	0	0	0	0
Appalachian Basin	1	0	0	0	0	0	0	0	0	0	0	0	0	0	0	0	0	0	0	0	0	0	0	0	0	0	0	0	0	0	0	0	0	0	0	0	0	0
Central Basin	0	0	0	0	1	0	0	0	0	0	0	0	0	0	0	0	1	0	0	0	0	0	0	1	0	0	0	0	0	0	0	1	0	0	1	0	0	0
Cincinnati Basin	0	0	0	1	0	0	0	0	0	0	0	0	0	1	0	0	0	0	0	0	0	0	0	0	1	1	1	1	1	1	1	1	1	1	1	1	1	1
Anticosti Island	0	0	0	0	0	0	0	0	0	0	1	1	1	1	1	0	0	0	0	0	0	0	0	0	0	0	1	0	0	0	0	0	0	0	0	0	0	0
N. Midcontinent	0	0	0	0	1	1	1	1	1	1	1	0	0	0	0	1	1	1	1	1	1	1	1	1	0	0	0	0	0	0	0	0	0	0	0	1	1	2
S. Midcontinent	2	1	1	1	1	0	0	1	0	0	0	0	0	0	0	0	1	0	0	0	0	0	0	0	0	0	0	0	0	0	0	0	0	0	0	1	2	1

Geodispersal

	22	23	24	25	26	46	47	48	49	50	51	52	53	54	55	56	57	58	59	60	61	62	63	64	67	68	69	73	74	75	76	77	78	79	80	81	82	83
Ancestor	0	0	0	0	0	0	0	0	0	0	0	0	0	0	0	0	0	0	0	0	0	0	0	0	0	0	0	0	0	0	0	0	0	0	0	0	0	0
Appalachian Basin	0	0	0	0	0	0	0	0	0	0	0	0	0	0	0	0	0	0	0	0	0	0	0	0	0	0	0	0	0	0	0	0	0	0	0	0	0	0
Central Basin	0	0	0	0	2	0	0	0	0	0	0	0	0	0	0	0	2	0	0	0	0	0	0	2	0	0	0	0	0	0	0	2	0	0	2	0	0	0
Cincinnati Basin	0	0	0	2	0	0	0	0	0	0	0	0	0	2	0	0	0	0	0	0	0	0	0	0	1	1	1	1	1	1	1	1	1	1	1	1	0	0
Anticosti Island	0	0	0	0	2	0	0	0	0	0	2	1	1	1	1	0	0	0	0	0	0	0	0	0	0	0	2	0	0	0	0	0	0	0	0	0	0	0
N. Midcontinent	0	0	0	0	0	1	1	1	1	1	1	0	0	0	0	1	1	1	1	1	1	1	1	1	0	0	0	0	0	0	0	0	0	0	0	2	0	1
S. Midcontinent	1	1	1	1	1	0	0	2	0	0	0	0	0	0	0	0	2	0	0	0	0	0	0	0	0	0	0	0	0	0	0	0	0	0	0	2	1	0
